# Structural basis for catalytic mechanism of human phosphatidylserine synthase 1

**DOI:** 10.1038/s41421-025-00775-3

**Published:** 2025-03-06

**Authors:** Yingjie Ning, Ruisheng Xu, Jie Yu, Jingpeng Ge

**Affiliations:** 1 School of Life Science and Technology, ShanghaiTech University, Shanghai, China; 2 Lingang Laboratory, Shanghai, China; 3 Interdisciplinary Research Center on Biology and Chemistry, Shanghai Institute of Organic Chemistry, Chinese Academy of Sciences, Shanghai, China; 4 University of Chinese Academy of Sciences, Beijing, China; 5 Shanghai Key Laboratory of Aging Studies, Shanghai, China

**Keywords:** Electron microscopy, Lipid signalling

Dear Editor,

Phosphatidylserine (PS) is a minor component of most biological membranes but plays critical roles in numerous cellular processes such as enzyme recruitment and activation, apoptosis, viral internalization and cholesterol trafficking^1–5^. In mammals, PS is synthesized de novo by two endoplasmic reticulum (ER) transmembrane enzymes, phosphatidylserine synthase 1 (PSS1) and phosphatidylserine synthase 2 (PSS2). These enzymes catalyze a calcium-dependent base-exchange reaction, substituting the head groups of phosphatidylcholine (PC) or phosphatidylethanolamine (PE) with l-serine^6^. While PSS2 specifically converts PE to PS, PSS1 utilizes both PC and PE as substrates. Mice lacking both PSS1 and PSS2 are lethal, and mutations in PSS1 that increase PS production lead to Lenz-Majewski syndrome (LMS)^7^. Furthermore, PSS1 inhibition can suppress the growth of PSS2-deficient cancer cell lines or B-cell lymphoma and increase low-density lipoprotein (LDL) expression and uptake^8–10^, suggesting its potential as a drug target for cancer treatment and blood cholesterol reduction. Despite the discovery of the base-exchange reaction for mammalian PS synthesis over 65 years ago and extensive functional characterizations since then, the molecular mechanisms underlying this process remain poorly understood. Here we determined cryo-EM structures of full-length human PSS1 in apo (PSS1^apo^), Ca^2+^-bound (PSS1^Ca^) and Ca^2+^/l-Serine-bound (PSS1^ser^) states, revealing how Ca^2+^, l-serine and an endogenous PC molecule are positioned in proximity to the negatively charged pocket to facilitate the base-exchange reaction. Furthermore, we identified eight pairs of lipid molecules bound to the PSS1 dimer, suggesting that lipids play crucial roles in PS synthesis.

We recombinantly expressed human full-length PSS1 with a C-terminal 3 C protease site, an enhanced green fluorescent protein (eGFP) and a Strep-tag II in HEK293S GnTI^−^ cells^11^. Based on detergent screening for membrane solubilization and gel filtration using fluorescence-detection size-exclusion chromatography (FSEC), we performed whole-cell solubilization with Lauryl Maltose Neopentyl Glycol/cholesteryl hemisuccinate (LMNG/CHS) and the subsequent purification with Glyco-diosgenin (GDN) (Supplementary Fig. S1a). To assess the enzymatic activity, we employed a radioactivity-based assay with proteoliposomes composed of the purified PSS1 and 1-palmitoyl-2-oleoyl-glycero-3-phosphocholine (POPC). The recombinant PSS1 exhibited robust PS synthase activity (Fig. 1a), comparable to previous results from cell homogenates and purified PSS1^6,12^, indicating that this recombinant PSS1 used for structural study is functional and can recapitulate native PSS1 function. We subsequently determined the cryo-EM structures of PSS1^apo^, PSS1^Ca^ and PSS1^ser^ in detergent micelles at overall resolutions ranging from 2.9 to 3.2 Å (Fig. 1b; Supplementary Figs. S1–S3 and Table S1). All these structures revealed eight endogenous lipid molecules per protomer, comprising five PC or PE molecules, two PS molecules (PS1 and PS2), and one phosphoinositide (PI), based on cryo-EM densities and the electrostatic features of the binding sites (Fig. 1b, c; Supplementary Figs. S2 and S4a, b). Given the similar shape and charge properties of PC and PE, along with their abundance in ER membranes, we could not definitively distinguish between them and therefore tentatively termed the five PC/PE-like molecules, including the one in the catalytic site, as PC molecules (PCs 1–5).

**Fig. 1:**
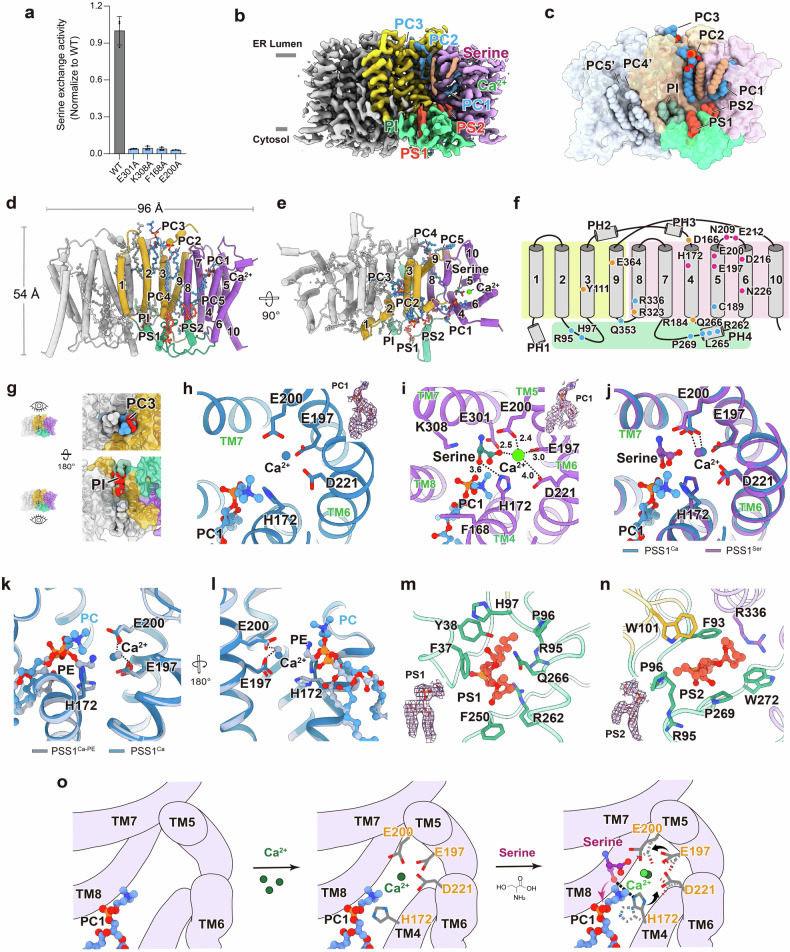
Functional and structural analysis of human PSS1. **a** Base-exchange activity of wild-type and mutant PSS1. Data are means ± SD (*n* = 3). **b** Cryo-EM map of PSS1^ser^. Densities for endogenous lipids bound to PSS1 are labeled. **c**–**e** Overall structure of PSS1. Surface and dot model of PSS1 shows the overall binding of lipids to PSS1 (**c**). Cylinder representation of PSS1 in side view parallel to membrane (**d**) and top-down view perpendicular to membrane (**e**). Transmembrane helices, Ca^2+^, l-serine, and lipid molecules are annotated. **f** Topology model of PSS1. Mutations identified in current and previous studies were annotated. Red, residues that affect enzymatic activity; blue, residues that affect PS regulation; orange, residues that affect PSS1 expression and stability. **g** Overview structure of lipids bound to dimer interface viewed from ER lumen side (top) and cytosolic side (down). **h**–**l** Structural details and comparisons of the catalytic site. Catalytic site in PSS1^Ca^ structure (**h**). Catalytic site in PSS1^ser^ structure (**i**). Structural comparison in the catalytic site between PSS1^Ca^ and PSS1^ser^ structures (**j**). Structural comparison in the catalytic site between PSS1^Ca^ and PSS1^Ca-PE^ (PDB: 9B4E) structures (**k**, **l**). **m**, **n** Structural details of PS binding site 1 (**m**) and PS binding site 2 (**n**). **o** Catalytic mechanism of PSS1. In the absence of Ca^2+^, PC binds to the catalytic site. The binding of Ca^2+^ stabilizes the pocket via E197, E200 and D221. Upon l-serine binding, the side chains of E200 and H172, along with Ca^2+^, shift toward l-serine to facilitate interactions. H172 subsequently protonates l-serine, enabling the exchange of its hydroxyl group with the headgroup of the bound PC or PE molecule.

PSS1 adopts a homodimeric assembly with dimensions of ~100 *×* 75 *×* 50 Å (Fig. 1d, e). Each protomer features 10 transmembrane helices (TMs 1–10), with both the N- and C- termini oriented toward the cytosolic side, and four peripheral α-helices (PHs 1–4) positioned at the transmembrane boundary near the EM lumen or cytoplasm (Fig. 1f). The structure can be categorized into three functional domains: (1) the scaffold domain (TMs 1–3 and 9), which interacts extensively with its counterpart in the opposing protomer to form the dimer interface; (2) the core domain (TMs 4–8 and 10), which constitutes the catalytic center of both protomers, positioned ~65 Å apart and oriented toward the ER lumen; and (3) the PS-binding domain, comprising PH4 and adjacent loops, which create a membrane-parallel platform that accommodates two PS molecules. The dimer interface, mediated by the scaffold domain, is further stabilized by two pairs of lipid molecules (Fig. 1g). On the ER lumen side, a pair of PC3 molecules interact with TM2 and TM3 from both protomers, with their acyl chains extending into a cavity and their phosphate groups interacting with PH2 (Supplementary Fig. S4c). On the cytosolic side, two PI molecules insert into a cavity formed by the scaffold and PS-binding domains (Supplementary Fig. S4d). Their hydrophobic tails align with the membrane leaflet, while their head groups are deeply embedded within the dimer interface. The extensive interactions between PSS1 and lipids suggest the potential roles of these lipids in maintaining the dimer formation and function of PSS1.

Recent studies have provided insights into the base-exchange mechanism by PSS1 structures complexed with Ca^2+^ and a PE molecule in the absence of l-serine^10^, yet structural basis for substrate recognition remains unclear. In the structures of PSS1^apo^, PSS1^Ca^ and PSS1^ser^, we observed a PC molecule (PC1) positioned within the catalytic core, between the halve created by TM4 and TM8 on the lipid leaflet side (Fig. 1h, i). This suggests that PC binds to the catalytic center independently of l-serine or Ca^2+^. Residue F168 on TM4 forms hydrophobic interactions with both TM8 and PC1, effectively separating the two cavities: one from the lipid leaflet side and the other from the ER lumen side, thus regulating access to the catalytic core. Mutation of F168 to alanine abolishes the PSS1 activity (Fig. 1a), possibly by disrupting the PC binding or accessibility. In the PSS1^Ca^ structure, we observed a Ca^2+^ in the catalytic center and surrounded by acidic residues including E197, E200 and D221 (Fig. 1h). The imidazole side chain of H172 directly interacts with the phosphoester group of PC1 at a distance of 2.9 Å. These interactions are not observed in the PSS1^apo^ structure when Ca^2+^ is absent, because of the less well-resolved local densities for the headgroup of PC1 and the sidechain of H172, despite the similar overall map quality. This suggests that Ca^2+^ helps to stabilize the negatively charged catalytic center. In the PSS1^ser^ structure, when both Ca^2+^ and l-serine are present, we observed that l-serine bound to the catalytic core near TM5 and TM7, with its carboxylic group interacting with Ca^2+^, its amino group interacting with the carboxylic group of E200 and its hydroxyl group interacting with the imidazole side chain of H172 (Fig. 1i). Alanine mutations of these residues have been shown critical for PSS1 catalytic function^12^. To further pinpoint residues essential for l-serine binding and PSS1 activity, we analyzed charged residues near l-serine and found that E200A, E301A and K308A disrupts PSS1 function without affecting its expression (Fig. 1a, h; Supplementary Fig. S1). E200 directly interacts with both l-serine and Ca^2+^, while E301 is located near the middle of the lipid bilayer and K308 resides at the ER lumen entrance to the catalytic pocket. These residues likely contribute to the binding and stabilization of l-serine which is critical for PSS1 function. Comparing the PSS1^Ca^ and PSS1^ser^ structures, the conformation of the TM helices is almost identical, while Ca^2+^ and the sidechains of substrate-interacting residues, such as H172 and E200, shift toward l-serine in the PSS1^ser^ structure (Fig. 1j). These structural transitions likely facilitate the protonation of l-serine by H172. Taken together, these structures suggest a possible base-exchange mechanism: a Ca^2+^ stabilized, negatively charged catalytic center attracts l-serine and PC or PE via their positively charged headgroups, allowing the hydroxyl group of l-serine, protonated by H172, to attack the phosphoester group of PC or PE, resulting in PS formation.

In our current and the complementary structural studies of human PSS1^10^, a PC and a PE molecule were built, respectively, according to the local cryo-EM densities and the features of the cavity. Structural alignment between our PSS1^Ca^ structure and their PSS1 structure (termed PSS1^Ca-PE^, PDB: 9B4E) showed substantial conformational transitions in the core domain in one protomer when aligned with the other, with an RMSD value of 1.86 Å over 377 Cα atoms (Supplementary Fig. S5). In the catalytic core, while the phosphate groups of PC and PE are positioned in a similar place, the choline and ethanolamine headgroups adopt different orientations, accompanied by the imidazole group of H172 (Fig. 1k, l). This is unlikely to be artifacts of model building, because densities for the sidechain of H172 are well-resolved in both studies and would not accommodate the alternative conformations. We speculate the different binding mode of PC and PE, along with conformational changes observed in the two studies, may result from differences in purification procedures and buffers. In the PSS1^Ca-PE^ structure, the protein was solubilized from membrane fractions while in our current PSS1^Ca^ structure, whole-cell solubilization was performed, which may have led to differences in lipid composition present in the final cryo-EM sample. The densities for PC and PE in both studies may represent mixtures of these lipids; nevertheless, these structural studies together provide insights into the substrate recognition and selection of PC and PE by PSS1.

Lipids are critical for PSS1 function, not only serving as substrates, but also regulating its activity. PS on the cytosolic leaflet of ER has been shown to inhibit PSS1^6^. In our structures of PSS1, we observed two PS-binding cavities formed by PH4 and nearby loops (Fig. 1c; Supplementary Fig. S4e). These sites are enriched with ring-containing residues, which facilitate hydrophobic interactions with the PS lipids. The head group of PS1 interacts with hydrophilic residues R262, R95, and Q266, while PS2 is positioned between the positively charged residues R95 and R336 (Fig. 1m, n). Alanine mutations of residues R95, H97, R262, Q266, and R336 individually remove PS inhibition and result in PS overproduction^12^. Similar effects have been observed in three gain-of-function mutations associated with Lenz-Majewski syndrome (LMS) (L265P, P269S, and Q353R)^7^. These mutations may alter the local structure of the PS-binding domain, thereby disrupting the PS binding and inhibition, leading to PS overproduction. Moreover, our cryo-EM structures reveal three PC molecules near the scaffold and core domains (PC2, PC4, and PC5) (Supplementary Fig. S4f, g). Among these, PC2, located in the ER lumen leaflet near TM3 and TM8, helps stabilize the interaction between these two domains. PC4 and PC5 found in the ER cytosolic leaflet, fill a cavity formed by the scaffold and core domains. Notably, one acyl chain of PC5 inserts between TM7 and TM8 of the core domain, potentially contributing to the stability of the catalytic center (Supplementary Fig. S4f). Moreover, the substrate PC1 directly interact with the inhibitor PS2 at their distal parts of the acyl chains (Supplementary Fig. S4). Taken together, the eight pairs of phospholipids identified in this study form a comprehensive protein–lipid interaction network (Supplementary Fig. S4a, b). The ER-facing surface created by the scaffold, core and PS-binding domains harbors multiple lipid-binding cavities, including those for the catalytic center (PC1) and PS binding site (PS1 and PS2), which may function as a lipid transfer “hub” to facilitate lipid binding or transfer.

In summary, we elucidated the cryo-EM structures of human PSS1 in various substrate-bound states, providing critical structural insights into its catalytic mechanism (Fig. 1o). A Ca^2+^ ion binding to a negatively charged pocket stabilizes the catalytic core, where the catalytic residue H172 facilitates the base-exchange reaction by interacting with both l-serine and the headgroup of PC1, protonating the hydroxylic group of l-serine to drive the reaction. Two PS molecules bound to the PS-binding domain likely inhibit PSS1 activity by locking it in an inactivated conformation. Additionally, a lipid-binding surface is identified in the PSS1 structure, suggesting its potential role in lipid binding and transfer. Our structural findings well explain previous biochemical characterizations of PSS1 and provide a foundation for understanding disease-associated mutations and guiding the development of drugs targeting PSS1-related diseases.

## Supplementary information

Supplementary Information

## Data Availability

The 3D cryo-EM density maps of PSS1^apo^, PSS1^Ca^, and PSS1^ser^ have been deposited in the Electron Microscopy Data Bank (EMDB) under the accession numbers EMD-62503, EMD-62506 and EMD-62505, respectively. Their atomic coordinates have been deposited in the Protein Data Bank (PDB) under the accession numbers 9KQF, 9KQJ, and 9KQI, respectively.
