# Supplementary material for: Structural basis for catalytic mechanism of human phosphatidylserine synthase 1
Source: Cell Discov. 2025 Mar 6;11:20. doi: 10.1038/s41421-025-00775-3 (PMC11882778; doi:10.1038/s41421-025-00775-3)
Supplement: Supplementary file 1 — Supplementary Information [file 41421_2025_775_MOESM1_ESM.pdf]

Supplementary Materials for

Structural basis for catalytic mechanism of human phosphatidylserine synthase 1

Yingjie Ning[1,2#], Ruisheng Xu[3,4#], Jie Yu[3, 5*], Jingpeng Ge[1*]

[1]School of Life Science and Technology, ShanghaiTech University, Shanghai, China.

[2]Lingang Laboratory, Shanghai, China.

[3]Interdisciplinary Research Center on Biology and Chemistry, Shanghai Institute of Organic Chemistry, Chinese Academy of Sciences, Shanghai, China.

[4]University of Chinese Academy of Sciences, Beijing, China.

[5]Shanghai Key Laboratory of Aging Studies, Shanghai.

#These authors contributed equally to this work.

*Corresponding author Email: gejp@shanghaitech.edu.cn.

This file includes:

Materials and Methods

Additional references

Supplementary Fig. S1 to S5

Supplementary Table S1

Materials and Methods

Cloning of hPSS1 cDNA

To amplify the hPSS1 cDNA fragment, a forward primer CGCGGAATTCACTAGTGCCACCATGGCGTCCTGCGTGGGGAGCCGGAC and a reverse primer CCTGGAACAGAACTTCCAGTTTCTTTCCAACGCCATTGGTG were used for PCR. The PCR product was cloned into the pEG BacMam vector with a C-terminal 3C protease cleavage site, an enhanced green fluorescent protein (eGFP) and a Strep II tag. The construct was verified by sequencing.

Cell culture and BacMam virus generation

Mammalian HEK293S GnTI$^-$ cells were grown in FreeStyle 293 Expression Medium supplemented with 1% (v/v) Fetal bovine serum (FBS) at 37 °C and 8% CO_2. The hPSS1 BacMam virus was generated and amplified in SF9 cells cultured in Sf-900 III SFM medium at 27 °C using the Bac-to-Bac baculorvirus system. In short, hPSS1 plasmid was transfected into DH10Bac to obtain bacmid. The bacmid was transfected into SF9 cells using Cellfectin and cultured for 5 days to generate P1 virus. Subsequently, P1 was added to SF9 cells at a 1:1000 (v/v) ratio and cultured for 5 days to obtain P2 virus. Both cell lines are routinely tested for mycoplasma contamination using CELLshipper Mycoplasma Detection Kit M-100 from Bionique and are mycoplasma free. No misidentified cell lines were used.

Expression and purification of hPSS1

A total of 800 ml of HEK293S GnTI$^-$ cells, at a density of 3 x 10^6 cells per ml, were infected with 80 ml of P2 virus. After 12 hours of infection, cells were supplemented with 10 mM sodium butyrate, shifted to 30 °C, and cultured for an additional 48 hours. Cells were collected by centrifugation, frozen in liquid nitrogen, and stored at -80 °C until use.

For purification, the harvested cells were lysed in buffer A containing 20 mM Tris pH 7.5, 150 mM NaCl, 1% (w/v) Lauryl Maltose Neopentyl Glycol (LMNG), 0.2%

(w/v) cholesteryl hemisuccinate (CHS), 5 mM $CaCl_2$, 1 mM phenylmethylsulfonyl fluoride (PMSF), 0.8 μM aprotinin, 2 μg/ml leupeptin and 2 μM pepstatin A for 2 hours at 4 °C. After centrifugation at 50,000 g for 1 hour to remove insoluble material, the supernatant was filtered through 0.45 μm filters and incubated with Strep-Tactin resin at 4 °C for 1 hour. The resin was washed extensively with buffer B containing 20 mM Tris pH 7.5, 150 mM NaCl, 0.01% (w/v) Glyco-diosgenin (GDN), 5 mM $CaCl_2$, and eluted with buffer B plus 5 mM desthiobiotin. The eluate was concentrated, incubated with 1:20 (w/w) 3C protease on ice for 1 hour to remove the C-terminal eGFP-Strep tag and further loaded onto a size-exclusion chromatography (SEC) column (Superose 6 Increase 10/300 GL) pre-equilibrated with buffer B. The peak fractions were collected and concentrated to 4.68 mg/ml for cryo-EM grid preparation or proteoliposome reconstitution. For PSS1 in the Ca^{2+} and L-serine-free state, $CaCl_2$ was excluded during protein purification.

Proteoliposome reconstitution and base-exchange activity assay

To prepare liposome, POPC was dissolved in chloroform, dried by a rotary evaporator, and placed in a vacuum chamber overnight. Dried lipid was dissolved by repeated freeze-thaw cycles and sonication in reconstitution buffer containing 20 mM Tris pH 7.5, 150 mM NaCl to a final concentration of 10 mg/ml. Lipids were extruded through 400 nm, 200 nm, and 100 nm filters using an Avanti Mini-Extruder, with each filter extruded 11 times. The extruded liposomes were divided into two equal portions: one half served as the empty liposome control, while the other half was used for reconstitution of proteoliposome. For proteoliposome reconstitution, the purified hPSS1 protein and lipids were mixed at a protein-to-lipid ratio of 1:20 (w/w) in the presence of 0.1% DM for 1 hour at 4 °C. For empty liposome control, all conditions remained the same except the volume of protein was replaced with SEC buffer. The mixtures were further loaded onto Sephadex G-50 resin pre-incubated by reconstitution buffer to remove detergent. Fractions containing proteoliposomes were collected and concentrated for functional assay.

hPSS1-POPC proteoliposomes were incubated with assay buffer containing 20

mM Tris pH 7.5, 150 mM NaCl, 5 mM $CaCl_2$, 200 μM L-serine and 400 nM [^{3}H] L-serine at 37 °C for 90 min. After stopping the reaction by adding 10 mM EDTA, the liposomes were loaded onto a nitrocellulose filter, washed twice with TBS, and dissolved in scintillation solution for radioactivity measurement.

Cryo-EM grid preparation, data collection and processing

A 4 μL aliquot of 4.68 mg/ml hPSS1 protein sample was applied to a glow-discharged Quantifoil 2/1 200 mesh Cu holey carbon grid. For L-serine-hPSS1 complex, 1 mM L-serine was incubated with hPSS1 protein on ice for 40 min before freezing grids. Grid preparation was performed using a Vitrobot IV with a wait time of 8 s, a blot time of 3 s and a blot force of 0 under 100% humidity at 4 °C, followed by rapid freezing in liquid ethane cooled by liquid nitrogen.

Data collection was performed using a 300 kV FEI Titan Krios equipped with a Gatan K3 camera and an energy filter. Movies were collected with a dose rate of ~20 e^-/pix/s, a total dose of 48 $e^-/Å^2$ and a defocus range of -1.0 to -2.0 μm. Movies were recorded in super-resolution mode with a pixel size of 0.5275 Å.

All data processing was done in cryoSPARC v4.6.0 unless otherwise mentioned[1]. For $PSS1^{Ca}$ state, 5500 raw movies in super-resolution mode were bin-2 motion-corrected with the patch motion correction. Contrast transfer function (CTF) values were calculated with the patch CTF estimation. Micrographs with CTF values over 5.5 Å were discarded. Particles were initially picked with blob picker. Bin-4 particles were extracted with a box size of 60 and were applied to 2 rounds of 2D classification. Good 2D class averages were selected and used for the templates for further template picking and Topaz particle picking. Following similar particle extraction and 2D classification strategies, particles from good 2D classes were combined with duplicates removed. Bin-2 particles were then re-extracted, followed by four rounds of triplicate ab-initio to generate an initial model. Particles were cleanup by heterogeneous refinement. Bin-1 particles were re-extracted refined by multiple rounds of heterogeneous refinement using C1 symmetry and non-uniform (NU) refinement using C2 symmetry, resulting in

the resolution to 3.14 Å. The particle stack was further subjected to local CTF refinement and local refinement with C2 symmetry, improving in a 2.95 Å.

For PSS1^{apo} or PSS1^{ser} states, data processing follow similar strategies as above, resulting in an overall reconstitution of 3.25 Å and 3.02 Å, respectively.

Model building

The initial model of PSS1 were predicted with AlphaFold2 and fitted into the cryo-EM maps in ChimeraX[2]. Models were manually adjusted in COOT[3]. Residues 1-13 aa at the N terminus, residues 409-373 aa at the C terminus, as well as residues 141-158 at the ER lumen side are missing due to the lack of cryo-EM densities. Five PC molecules, two PS molecules and one PI molecule were modeled to each protomer of PSS1 based on the features of cry-EM densities and the local structures. The L-serine and Ca^{2+} ion were built manually by comparing the differences in cryo-EM maps of PSS1 in various substrate-bound states. There models were refined in real space using Phenix[4]. All structure figures were prepared using ChimeraX[2].

Additional References

1. Punjani, A., Rubinstein, J.L., Fleet, D.J., and Brubaker, M.A. (2017). cryoSPARC: algorithms for rapid unsupervised cryo-EM structure determination. Nat Methods *14*, 290-296. 10.1038/nmeth.4169.
2. Goddard, T.D., Huang, C.C., Meng, E.C., Pettersen, E.F., Couch, G.S., Morris, J.H., and Ferrin, T.E. (2018). UCSF ChimeraX: Meeting modern challenges in visualization and analysis. Protein Sci *27*, 14-25. 10.1002/pro.3235.
3. Emsley, P., and Cowtan, K. (2004). Coot: model-building tools for molecular graphics. Acta Crystallogr D Biol Crystallogr *60*, 2126-2132. 10.1107/S0907444904019158.
4. Afonine, P.V., Poon, B.K., Read, R.J., Sobolev, O.V., Terwilliger, T.C., Urzhumtsev, A., and Adams, P.D. (2018). Real-space refinement in PHENIX for cryo-EM and crystallography. Acta Crystallogr D Struct Biol *74*, 531-544. 10.1107/S2059798318006551.

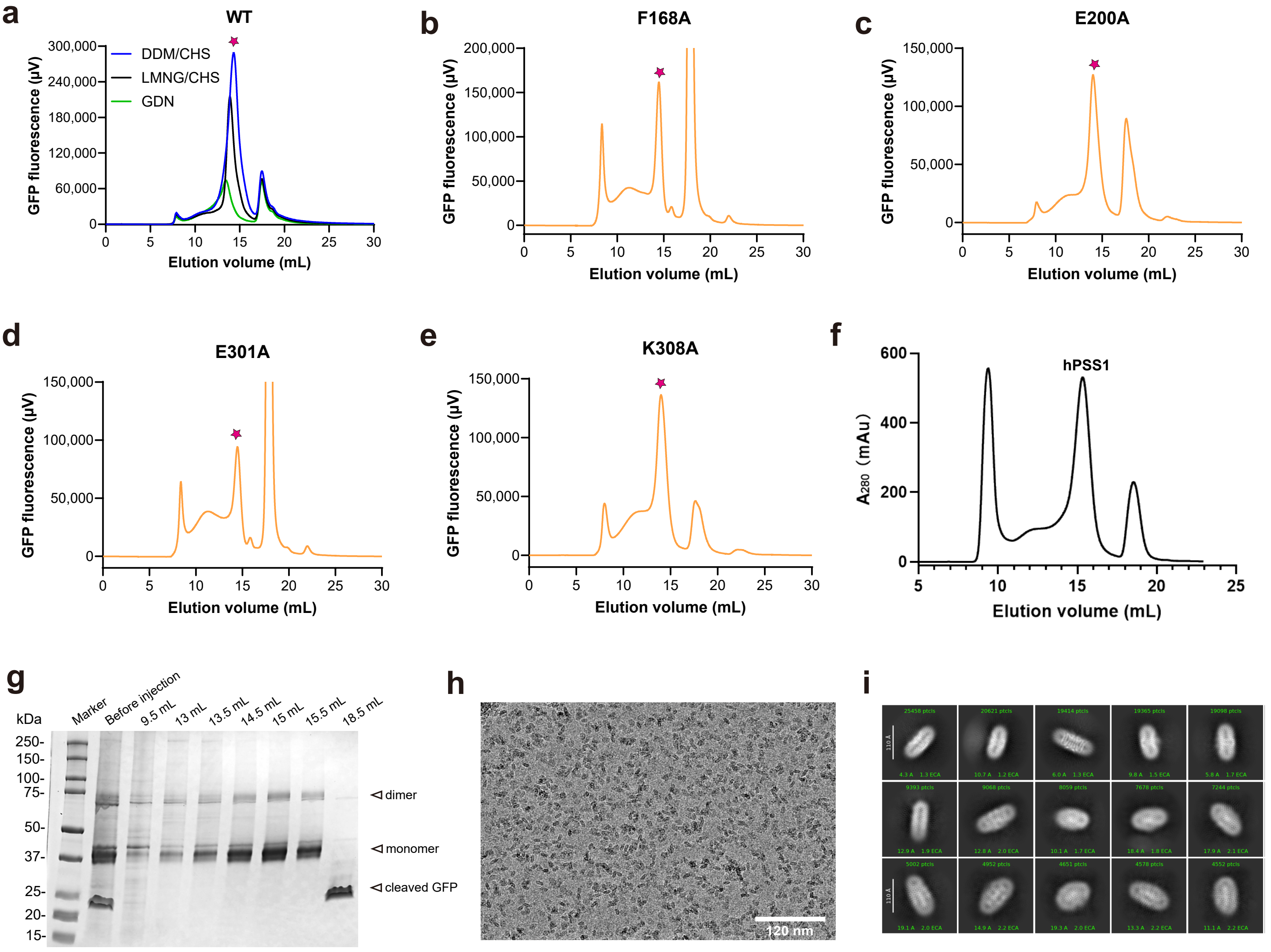

Supplementary Fig. S1 Biochemical and cryo-EM characterization of PSS1.

a Representative FSEC traces for detergent screening of PSS1. Cells solubilized in different detergents. The peak of PSS1 is indicated by a star.

b-e FSEC screening of PSS1 mutants. The peak of PSS1 is indicated by a star.

f Size exclusion chromatography of PSS1.

g SDS-PAGE gel of purified PSS1 after gel filtration.

h A representative cryo-EM micrograph of PSS1. Scale bar, 120 nm.

i Representative 2D averages. Scale bar, 110 Å.

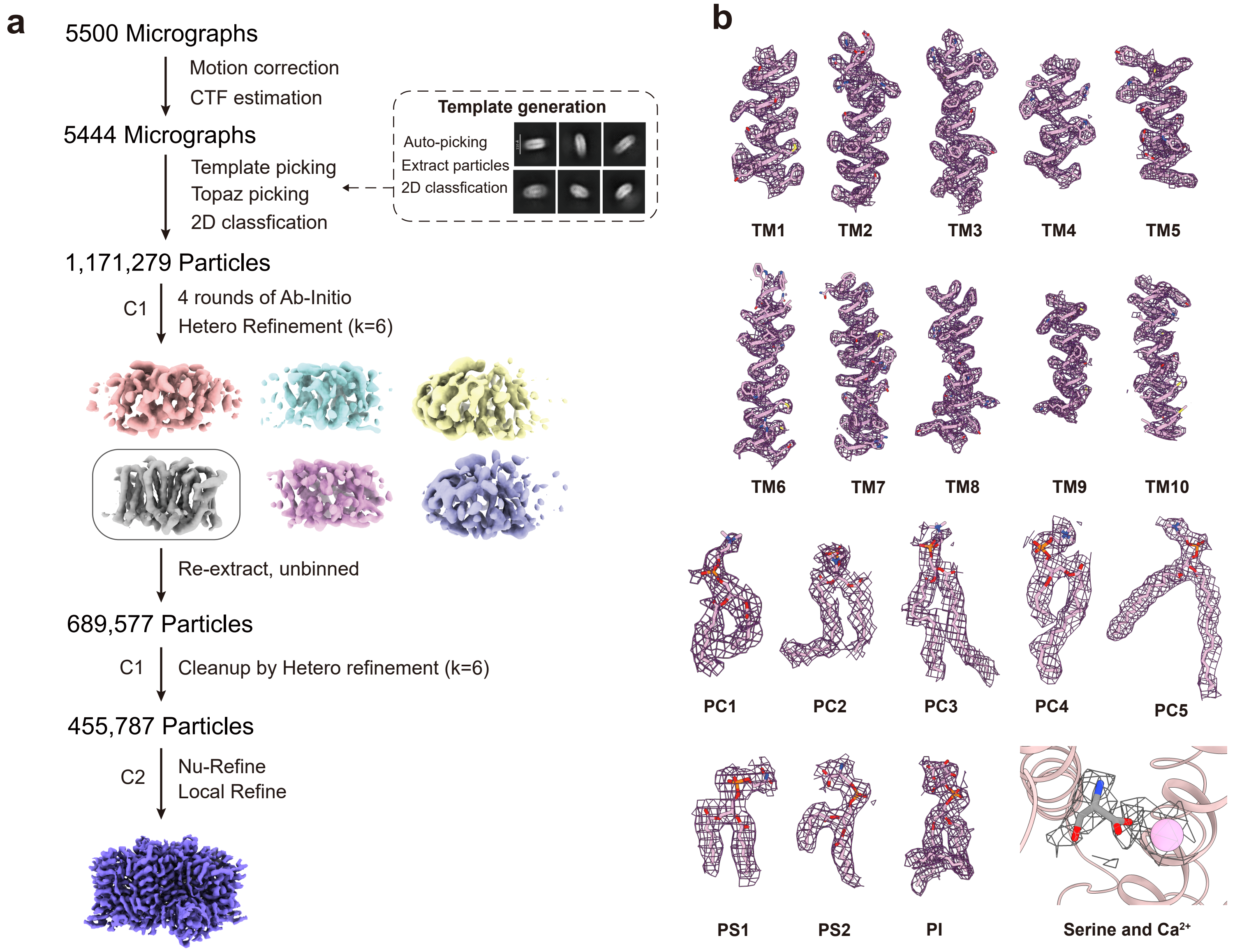

Supplementary Fig. S2 Data processing working flow and cryo-EM densities for transmembrane helices and endogenous bound lipids.

a Data processing working flow of PSS1^{Ca}. Details are provided in Methods.

b Cryo-EM densities of the transmembrane helixes, lipids, Serine and Ca^{2+}. Densities are shown as meshes.

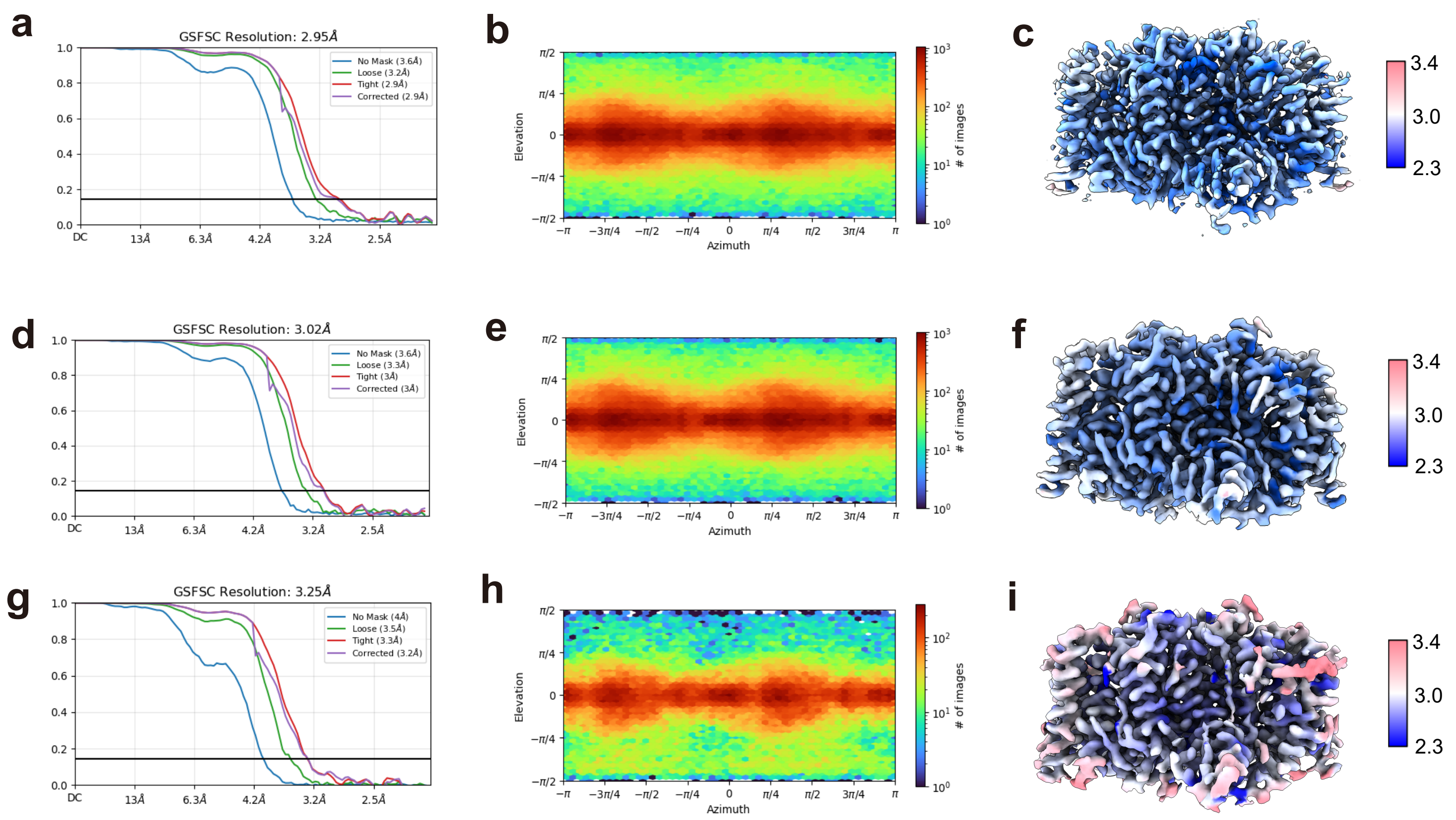

Supplementary Fig. S3 FSC curves, angular distribution and local resolution of cryo-EM maps.

a-c FSC curves, angular distribution and local resolution of PSS1^{Ca}.

d-f FSC curves, angular distribution and local resolution of PSS1^{ser}.

g-i FSC curves, angular distribution and local resolution of PSS1^{apo}.

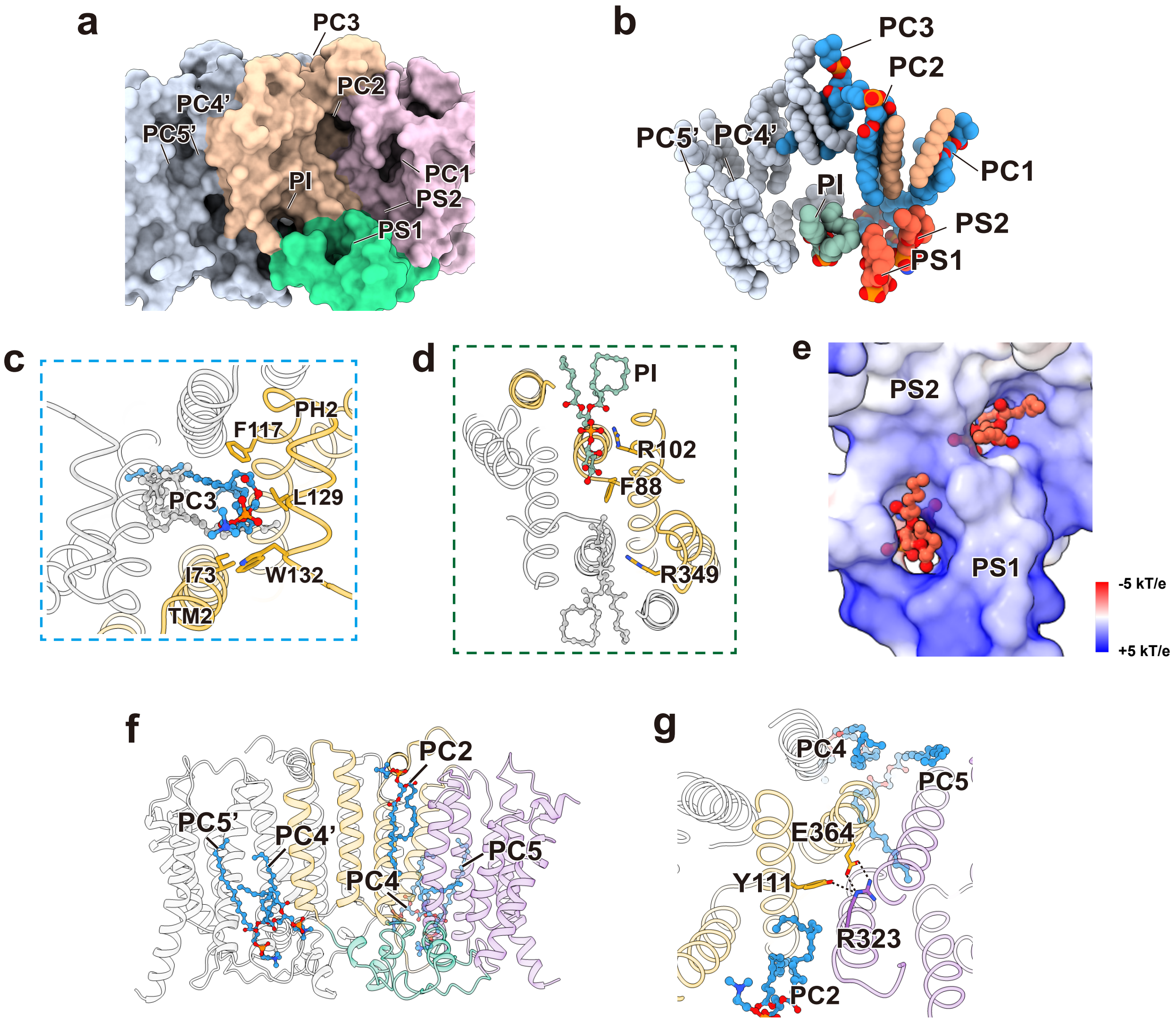

Supplementary Fig. S4 PSS1 interactions with lipids.

a Surface representation of PSS1 shows multiple cavities suitable for specific lipid binding. The scaffold domain is colored with yellow; the core domain is colored with pink and the PS-binding domain is colored with green.

b A sphere representation of the endogenous lipids observed in the cryo-EM structures of PSS1.

c, d Interactions at dimer interface, viewed from ER lumen side (c) and cytosolic side (d), respectively. **c** Structural details of the PC3 mediated dimer interface. **d** Structural details of the PI mediated dimer interface.

e Electrostatic potential surface shows two positively charged pocket for PS binding.

f, g Structural details and lipid interactions of the interface the scaffold and core domains. **f** Interactions between PC2, PC4, PC5 and PSS1.

g Residues crucial for PSS1 function positioned in the interface between the scaffold and core domains.

.

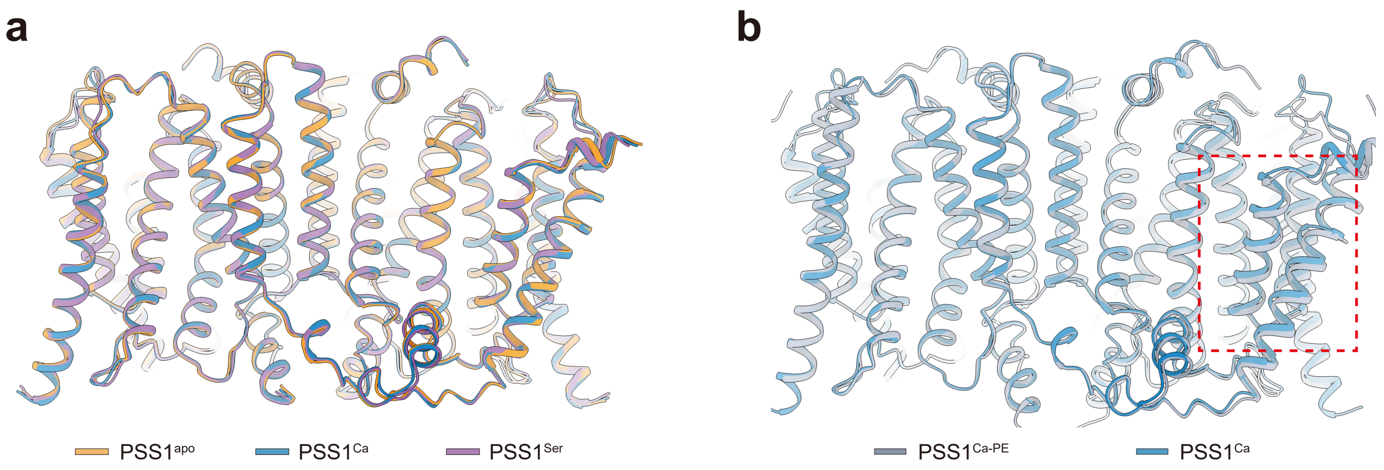

Supplementary Fig. S5 Structure alignments of PSS1.

a Structural alignment of PSS1^{apo}, PSS1^{Ca} and PSS1^{ser}. PSS1^{apo} is colored with orange; PSS1^{Ca} is colored with blue and PSS1^{ser} is colored with purple.

b Structural alignment of PSS1^{Ca} and PSS1$^{Ca\text{-}PE}$ (PDB: 9B4E). Structural difference at the core domain is labeled. PSS1^{Ca} is colored with blue and 9B4E is colored with grey.

Supplementary Table S1

	Apo-hPSS1 (EMD-62503), (PDB-9KQF)	Ca^{2+}-hPSS1 (EMD-62506), (PDB-9KQJ)	L-serine-hPSS1 (EMD-62505), (PDB-9KQI)
**Data collection and processing**			
Magnification	x81,000	x81,000	x81,000
Voltage (kV)	300	300	300
Electron exposure (e^{-}/Å^2)	48	48	48
Defocus range (μm)	-1.0 to -2.0	-1.0 to -2.0	-1.0 to -2.0
Pixel size (Å)	1.055	1.055	1.055
Symmetry imposed	C2	C2	C2
Initial particle images (no.)	834,418	1,171,279	1,244,619
Final particle images (no.)	109,125	455,787	446,795
Map resolution (Å)	3.25	2.95	3.02
FSC threshold	0.143	0.143	0.143
**Refinement**			
Initial model used (PDB code)			
Model resolution (Å)	3.4	3.2	3.2
FSC threshold	0.5	0.5	0.5
Model resolution range (Å)			
Map sharpening B factor (Å^2)	148.5	157.0	163.4
Model composition			
Non-hydrogen atoms	7066	7056	7082
Protein residues	754	754	754
Ligands	20	22	24
B factors (Å^2)			
Protein	27.12/160.97/79.41	11.56/127.02/54.59	5.77/141.08/57.81
Ligand	59.85/133.86/82.45	37.21/100.39/57.31	35.23/105.66/57.68
R.m.s. deviations			
Bond lengths (Å)	0.005	0.005	0.005
Bond angles (°)	0.618	0.623	0.557
**Validation**			
MolProbity score	1.85	1.94	1.49
Clashscore	11.57	9.29	9.25
Poor rotamers (%)	0.44	3.24	0.29
Ramachandran plot			
Favored (%)	95.98	97.72	98.12
Allowed (%)	4.02	2.28	1.88
Disallowed (%)	0	0	0
